# A Randomized Double-Blind Comparative Study of the Intubating Conditions and Hemodynamic Effects of Rocuronium and Succinylcholine in Pediatric Patients

**DOI:** 10.7759/cureus.44631

**Published:** 2023-09-04

**Authors:** Anil Kumar, Arvind Kumar, Alok K Bharti, Annu Choudhary, Mumtaz Hussain, Shashank Dhiraj

**Affiliations:** 1 Anesthesiology, Indira Gandhi Institute of Medical Sciences, Patna, IND; 2 Anesthesiology and Critical Care, Indira Gandhi Institute of Medical Sciences, Patna, IND

**Keywords:** rapid sequence induction, airways, paediartic patients, succinylcholine, rocuronium bromide, laryngoscopy, intubation

## Abstract

Background: The incidence of unanticipated difficult airways is higher in pediatric age groups than in adults due to the different airway anatomy, difficulty in airway examination, and congenital malformations. Rocuronium bromide has a comparable onset time to succinylcholine at its proportionate dose. Hence, we compared rocuronium bromide with succinylcholine to assess intubating conditions and their side effects, if any.

Method: A total of 200 pediatric patients of American Society of Anesthesiologists (ASA) grades I and II between one and 14 years of age of either sex posted for elective surgery were included in the study. After randomization, group R (n = 100) received 1.2 mg/kg rocuronium, and group S (n = 100) received 2 mg/kg succinylcholine intravenously. After confirming the mask ventilation, the study drugs were administered, and intubating conditions were assessed as excellent, good, poor, or impossible. Hemodynamic changes post-intubation were recorded as our secondary outcome.

Result: Intubating conditions were excellent( 65%), good( 25%) and fair (10%) in patients of group R, while results in group S were excellent( 60%), good( 20%), fair (15%), and poor (5%) (p = 0.010). The heart rate was significantly increased post-intubation in group S, while there was no significant increase in systolic or diastolic blood pressure in either group.

Conclusion: At a dose of 1.2 mg/kg body weight, rocuronium was a better alternative to succinylcholine for providing rapid intubating conditions and stable hemodynamics without associated adverse effects.

## Introduction

The anatomy and physiology of neonates and infants are different from those of adults, creating challenges for anesthesiologists. Among them, of particular concern is the airway. Paediatric airways can be classified as 'normal', 'impaired normal', and 'known abnormal' [1]. The incidence of unanticipated difficult airways is higher in pediatric age groups than adults due to different airway anatomy, difficulty in airway examination, and congenital malformations [2]. Securing the airway of a pediatric patient in the operating theater is a real challenge for anesthesiologists. Succinylcholine is most frequently used by anesthetists and is regarded as the gold standard for difficult tracheal intubation [3]. Controlled rapid-sequence intubation without cricoid pressure is a safe alternative for pediatric patients to manage a difficult airway [4]. The primary reason for succinylcholine's popularity is its ability to swiftly establish favorable intubating conditions, which improves safety by allowing early establishment of the patient's airway and hence reducing the risk of aspiration. While succinylcholine has a rapid onset and excellent intubating properties, it can have certain potentially dangerous adverse effects that range from modest patient discomfort (due to postoperative myalgia) to potentially fatal occurrences of arrhythmias and malignant hyperthermia. There are reports [5] of rare but life-threatening malignant hyperpyrexia, hyperkalaemia, and cardiac arrests in young boys with undiagnosed muscular dystrophy. It is contraindicated in patients with severe burns, crush injuries, severe abdominal infections, denervation syndromes, spinal cord injuries, and those with a history of malignant hyperthermia or an allergic reaction to it. So, the US Food and Drug Administration recommends that the use of succinylcholine in children should be reserved for emergency intubation and instances where immediate securing of the airway is necessary [6].

Rocuronium bromide is the first non-depolarizing agent to be accepted as an alternative to succinylcholine in emergencies requiring quick intubation. It has a faster onset time than any other non-depolarizing agent. Moreover, several researchers have stated that the time required to intubate the trachea was exactly proportionate to its dose. It is predicted to have a similar onset time to succinylcholine without the negative side effects. However, rocuronium has a similar duration of action as vecuronium bromide. Thus, it is undesirable in situations where a brief period of apnea is required. Reduced doses of neuromuscular blocking agents should theoretically result in a decreased duration of action [7]. Several researchers have compared rocuronium and succinylcholine at lower intubating doses in adult and pediatric patients. However, to the best of our knowledge, no one has studied the intubating conditions produced by these two muscle relaxants at higher doses in pediatric patients. Hence, we compared rocuronium bromide with succinylcholine to determine intubating conditions in pediatric patients. Post-intubation hemodynamic changes were also recorded as a secondary outcome.

## Materials and methods

This is a single-center, double-blinded (the intubating anesthesiologist and assistant injecting the study drug were blinded), randomized, comparative study conducted from May 2020 to January 2022. Ethical clearance was obtained from the Institutional Ethics Committee of the Indira Gandhi Institute of Medical Sciences (approval no. 1075/IEC/IGIMS/2019). The primary aim of our study was to assess the intubating conditions produced by rocuronium and succinylcholine in pediatric patients. The secondary objective of our study was to measure the hemodynamic response to laryngoscopy and intubation.

The sample size (n) was calculated according to the formula n = Z2* P* (1-P)/e2, where Z = 1.96 for a confidence level (α) of 95%, P = proportion (expressed as a decimal), and e = margin of error. Keeping Z = 1.96, P = 0.153, and e = 0.05, the sample size was calculated to be 200.

All the data were analyzed using SPSS Statistics version 26.0 (IBM Corp., Armonk, NY, USA). The data were presented as descriptive statistics for continuous variables and percentages for categorical variables and subjected to a chi-square test, Fischer's exact test, and t-test. Other values were represented in number, proportions (%), and mean ± SD.

A total of 200 pediatric patients aged between one and 14 years of either sex and of American Society of Anesthesiologists (ASA) grades I and II posted for elective surgery were included in the study. Exclusion criteria were patient refusal, hyperkalemia, malignant hyperthermia, neuromuscular disease, history of cardiac, renal or hepatic disorders, and patients receiving medication known to influence neuromuscular function or any known allergy to the study's drugs.

After obtaining written and informed consent from parents, cases were selected after a thorough pre-anesthetic assessment, including a detailed clinical history, examination, and relevant laboratory investigations. Selected patients were randomly divided into two groups of 100 patients each, based on computer-generated random numbers, through opaque-sealed envelopes. Group R received an injection of rocuronium 1.2 mg/kg IV, and group S received an injection of succinylcholine 2 mg/kg IV. The study's drugs, rocuronium 1.2 mg/kg IV and succinylcholine 2 mg/kg IV, were prepared by an independent anesthesiologist after opening the sealed envelope for the name of the study drug in a 5 ml syringe according to the weight of the patient. The final volume of the study drug was 5 ml in both groups.

In the operation theater, the standard ASA monitors, i.e., electrocardiogram, non-invasive blood pressure, and pulse oximeter, along with neuromuscular monitoring, were attached. A suitable peripheral intravenous cannula was placed into an available vein in the ward, and a venous fluid drip was started there. All children were premedicated with an injection of midazolam 0.01 mg/kg, an injection of glycopyrrolate 10 mcg/kg, and an injection of fentanyl 2 mcg/kg. After preoxygenation with 100% oxygen for 5 minutes, patients were induced with an injection of propofol 2 mg/kg. Confirming adequate mask ventilation, patients were given a study drug, and laryngoscopy was attempted at 45 seconds. The anesthesiologist was blinded to the study drug as he had his back turned for 45 seconds after the study drug was given. Laryngoscopy and intubation were attempted at the 45th second for every patient in both groups. Intubating conditions were assessed according to the four-point scale of Cooper et al. [6]. Individual scores were added to give overall intubation scores of 8-9 (excellent), 6-7 (good), 3-5 (fair), and 1-2 (poor) (Table 1).

**Table 1:** Scoring system

Score	Jaw relaxation	Vocal cords	Response to intubation
0	Poor (impossible )	Closed	Severe coughing/Bucking
1	Minimal (difficulty)	Closing	Mild cough
2	Moderate (fair )	Moving	Slight diaphragmatic movement
3	Good (easy )	Open	None

The time taken from when the muscle relaxant was injected to the maximum depression of twitch height was also recorded to calculate the onset time for the muscle relaxant used. The hemodynamic response to laryngoscopy and intubation was recorded for the first 5 minutes. Anesthesia was maintained with oxygen, nitrous oxide, and sevoflurane. Any intraoperative surgical complication or hemodynamic instability led to the exclusion of the patient from the final analysis. After the end of surgery, patients were reversed, extubated, and shifted to the post-anesthesia care unit for further monitoring.

## Results

**Figure 1:**
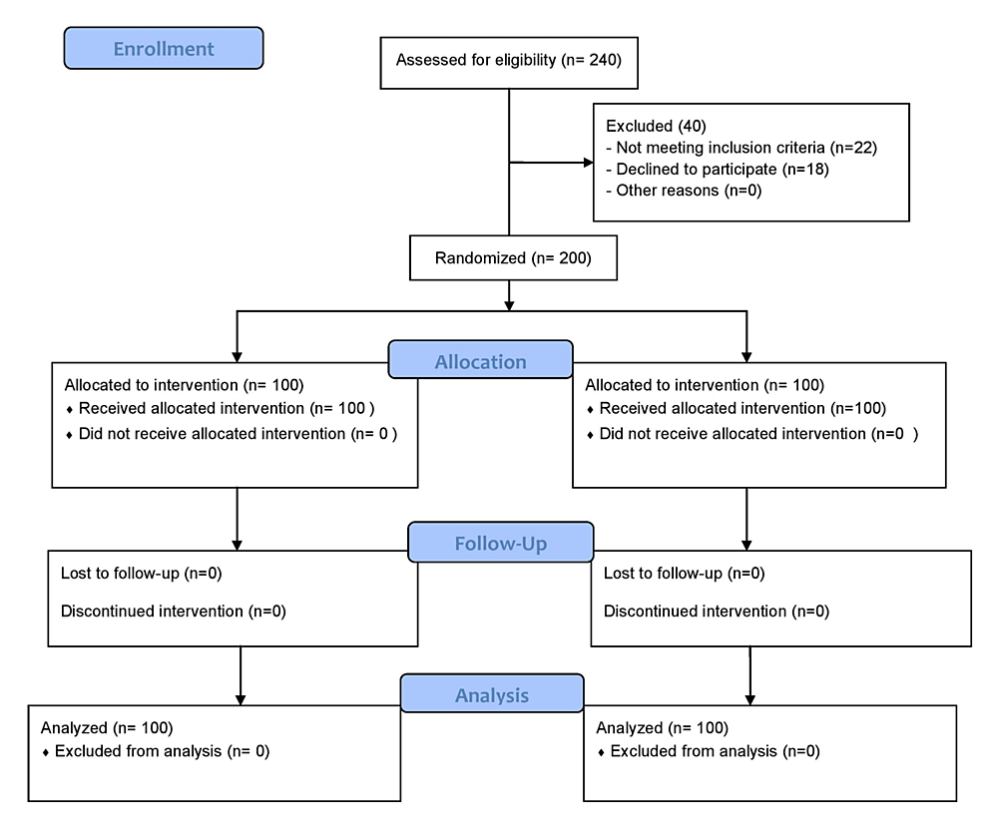
Consort flow diagram

Out of 240 patients, 200 completed the study successfully (Figure 1).

Demographic profiles were comparable in terms of age, sex, weight, and ASA physical status of patients as shown in Table 2.

**Table 2:** Demographic variables Data are presented as mean± SD with p <0.05 considered statistically significant. ASA: American Society of Anesthesiologists

Demographic variable	Group R (mean ± SD)	Group S (mean ± SD)	p-value
Age (years)	6.61±4.25	5.24±3.17	0.589
Weight (kg)	17.92±8.73	15.56±6.61	0.030
ASA grading	50.00±46.66	50.00±39.59	1.000
Sex (male/female)	87/13	88/12	0.83

It was observed that rocuronium (group R) produced better intubating conditions, which was statistically significant (p = 0.01) (Table 3). However, the intubating condition was either excellent or good in both groups.

**Table 3:** Comparison of intubating conditions between the two groups

Parameter	Group R	Group S	p-value
Excellent	65	60	0.010
Good	25	20	
Fair	10	15	
Poor	0	5	
Total	100	100	

Hemodynamically, patients were compared at various time intervals, such as baseline, after induction, after muscle relaxant, and after intubation at 0, 1, 3, and 5 minutes. All parameters were comparable at all points in time except heart rate (Tables 4-6).

**Table 4:** Comparison of mean diastolic blood pressure (mmHg) between the two groups Data are presented as mean± SD with p <0.05 considered statistically significant.

Diastolic blood pressure (mmHg)	Group R	Group S	t-test	p-value
	Mean ± S. D	Mean ± S. D		
Baseline	63.69±6.07	63.12±6.54	0.699	0.486
After induction	64.15±10.75	58.93±6.52	4.302	0.439
After muscle relaxant	62.32±10.13	56.69±5.03	5.153	0.397
0 minutes	67.02±7.84	60.24±6.25	6.976	0.528
1 minute	66.82±8.28	66.25±9.52	0.460	0.646
2 minutes	64.49±8.28	64.26±8.10	0.203	0.840
5 minutes	61.67±6.53	65.78±8.71	-3.869	0.618

**Table 5:** Comparison of mean systolic blood pressure (mmHg) between the two groups Data are presented as mean± SD with p <0.05 considered statistically significant.

Systolic blood pressure (mmHg)	Group R	Group S	t-test	p-value
	Mean ± S. D	Mean ± S. D		
Baseline	98.88±10.98	115.88±6.35	-14.010	0.328
After induction	102.04±21.20	107.85±16.62	-2.272	0.025
After muscle relaxant	99.66±17.64	100.27±5.57	-.334	0.739
0 minutes	104.73±17.54	101.41±5.34	1.824	0.071
1 minute	106.57±20.66	102.68±5.60	1.779	0.078
2 minutes	108.90±20.09	106.12±7.56	1.235	0.220
5 minutes	106.30±18.45	105.02±11.49	0.558	0.578

**Table 6:** Comparison of mean heart rate between the two groups Data are presented as mean± SD with p <0.05 considered statistically significant.

Heart rate (bpm)	Group R	Group S	t-test	p-value
	Mean ± S. D	Mean ± S. D		
Baseline	109.85±9.03	115.31±8.632	-5.412	<0.001
After induction	100.69±12.43	111.21±7.47	-8.758	<0.001
After muscle relaxant	98.58±8.29	103.65±8.66	-5.083	0.002
0 minutes	108.45±8.85	98.08±6.62	11.138	0.002
1 minute	113.16±6.08	107.97±11.54	4.326	0.060
2 minutes	109.55±6.15	105.94±9.15	3.642	0.038
5 minutes	110.82±7.47	104.17±8.01	6.268	0.534

As is known, fasciculation and myalgia occurred in the succinylcholine group but did not have any adverse effect on anesthesia or surgery. Three patients complained of vomiting in the rocuronium group, but none in the succinylcholine group. Overall, no significant or life-threatening side effects occurred in either group.

## Discussion

We found that rocuronium at a dose of 1.2 mg/kg body weight produced excellent intubating conditions at 45 seconds in pediatric patients. The observation might be a result of the higher intubating dose of rocuronium used in our study. Other researchers have also performed similar studies but with different doses in both adult and pediatric patients. Cheng et al. compared various doses of rocuronium with 1.5 mg/kg succinylcholine for intubating conditions in children aged one to 10 years old posted for elective surgery and found that rocuronium 0.9 mg/kg and succinylcholine 1.5 mg/kg body weight produced excellent intubating conditions at 60 seconds [8]. Barve et al. conducted a randomized, prospective study in 40 ASA grade I-II children aged one to five years with rocuronium 0.6 mg/kg or succinylcholine 1 mg/kg and observed no significant difference in intubating conditions or percentage of block in twitch height. With rocuronium, 13 patients (65%) could be intubated at 60 seconds and 20 (100%) at 90 seconds, while all were intubated at 60 seconds with succinylcholine [9]. These results are similar to ours, with the only difference being 60 seconds compared to our 45 seconds, which could be due to the use of low-dose rocuronium. Stoddart et al. conducted a blind, randomized study in 60 children undergoing elective tonsillectomy and compared intubating conditions at one minute with sccinylcholine 1.0 mg/kg and rocuronium 0.6 mg/kg. They found that there was no difference in the intubating conditions at one minute, with 25 excellent and five good scores in the sccinylcholine group and 27 excellent and three good scores in the rocuronium group [10]. These results are similar to our findings, but at 60 seconds, whereas we compared higher doses of both drugs at 45 seconds. Li et al. compared an intravenous bolus injection of 1 mg/kg of succinylcholine (n = 141) with 1.2 mg/kg of rocuronium (n = 126) for a rapid sequence induction in the emergency department and found no statistically significant difference in the number of patients with successful first-attempt orotracheal intubation between the two groups (112 vs. 87, p = 0.067) [11]. The findings of this study support our results, showing that even in an emergency setting, rocuronium was equally effective as succinylcholine in securing the airway at higher doses. Moreover, it is devoid of adverse effects like hyperkalemia and has a longer duration of action.

Tran et al. [12] performed a systemic review comparing rocuronium with succinylcholine to determine intubating conditions during rapid sequence intubation of the trachea. They found succinylcholine superior over rocuronium for achieving excellent intubating conditions (risk ratio (95% CI) 0.86 (0.81 to 0.92), n = 4151) and clinically acceptable intubation conditions (risk ratio (95% CI) 0.97 (0.95-0.99), n = 3992). Kumar et al. [13] carried out a trial on 80 adult cases requiring rapid sequence induction for emergency surgery. They concluded that rocuronium was a better choice in cases where succinylcholine was contraindicated. Wahid et al. [14] compared rocuronium (1 mg/kg) with succinylcholine (1 mg/kg) for rapid sequence induction under general anesthesia. They observed that both drugs were equally effective in terms of excellent intubation conditions for rapid sequence induction in patients undergoing emergency surgery. A large observational study [15] compared first-pass intubation success between patients receiving succinylcholine and those receiving rocuronium. First-pass intubation success rate was 87.0% with succinylcholine versus 87.5% with rocuronium (adjusted odds ratio: 0.9; 95% CI, 0.6 to 1.3). They did not find any association between paralytic choice and first-pass rapid-sequence intubation success or peri-intubation adverse events. A study [16] analyzed various doses of rocuronium for first-attempt success rates among emergency department patients undergoing rapid sequence intubation. They concluded rocuronium dosed at ≥1.4 mg/kg was associated with higher first-attempt success without any adverse events. This finding supports our study, as we used a higher dose of rocuronium for rapid securing of the airway in pediatric patients.

The hemodynamic response of both drugs was comparable at all time intervals except heart rate after giving muscle relaxants and was significantly higher in the succinylcholine group (p = 0.002) in our study. Tang et al. found that the heart rate was significantly accelerated in the 1.5 mg/kg succinylcholine group, whereas a mild effect was observed in the 1.0 mg/kg succinylcholine and 1.2 mg/kg rocuronium groups. At higher doses, succinylcholine increases anaerobic metabolism, disturbs the oxygen supply and demand balance, and increases the risk of hemoglobin desaturation [17]. We also found a significant increase in heart rate after injecting succinylcholine as compared to rocuronium, which settled by itself after a few minutes. Makhija et al. [18] studied the intubating condition and hemodynamic effect of rocuronium versus the rocuronium-vecuronium/pancuronium combination in pediatric cardiac patients and found that the rocuronium-vecuronium combination provided excellent intubating conditions with stable cardiovascular stability.

The major side effects in the succinylcholine group were fasciculation and myalgia, while none of the patients in the rocuronium group experienced these side effects. Thus, rocuronium at higher doses is a safe and effective muscle relaxant for securing airways in pediatric patients without significant side effects.

Limitations

Blinding was difficult in this study, as fasciculations can't be masked, but we kept our anesthesiologist with his back facing towards the patient and tried to mask the fasciculations. Intubating condition assessment might be variable between different observers as it would depend on the expertise and experience of the anesthesiologist. This could lead to observer bias.

## Conclusions

Rocuronium at a dose of 1.2 mg/kg is a better alternative to succinylcholine for providing rapid intubating conditions in pediatric patients. It provides a stable hemodynamic condition during laryngoscopy and intubation. And it does so without any significant adverse effects such as myalgia and fasciculation, which are commonly associated with succinylcholine.
